# Supplementary material for: Transcriptome Sequencing and Metabolome Analysis Reveal Genes Involved in Pigmentation of Green-Colored Cotton Fibers
Source: Int J Mol Sci. 2019 Sep 29;20(19):4838. doi: 10.3390/ijms20194838 (PMC6801983; doi:10.3390/ijms20194838)
Supplement: Supplementary file 1 [file ijms-20-04838-s001.zip › ijms-579638-supplementary/Supplementary Files/ijms-579638-Supplementary Figures.doc]

**Figure 1.** Plots from partial least squares discriminant analyses (PLS-DA) for modeling the differences between two cultivars at 12 (**A**), 18 (**B**) and 24 (**C**) days post- anthesis (DPA) using electrospray ionization (ESI^+^ and ESI^−^).

**Figure 2.** (**A**) Phenylpropanoid pathway gene transcripts. (**B**) Correlation of gene expression obtained from quantitative polymerase chain reaction (qPCR) analysis and RNA-Seq in green-colored fiber (GCF) and white-colored fiber (WCF).
